# Anti-neutrophil Cytoplasmic Antibody-Associated Vasculitis With Periaortitis That Developed After mRNA COVID-19 Vaccination

**DOI:** 10.7759/cureus.37480

**Published:** 2023-04-12

**Authors:** Yuta Yoshino, Takeshi Ishida

**Affiliations:** 1 Internal Medicine, Saitama Citizens Medical Center, Saitama, JPN

**Keywords:** periaortitis, microscopic polyangiitis, sars-cov-2, vaccine, covid-19

## Abstract

Severe acute respiratory syndrome coronavirus type 2 (SARS-CoV-2) has caused a global pandemic resulting in many deaths. As a result, vaccines to prevent the onset of coronavirus disease 2019 (COVID-19) have been developed and have demonstrated high efficacy in large-scale clinical trials. Adverse events that develop within a few days after vaccination are common, such as fever, malaise, body aches, and headaches, and have become widely known as transient reactions. However, as COVID-19 vaccines are administered worldwide, several studies have highlighted that long-term side effects associated with vaccines against SARS-CoV-2 may include serious adverse events. There has been an increase in reports of COVID-19 vaccinations being associated with the onset of autoimmune diseases, such as anti-neutrophil cytoplasmic antibody (ANCA)-associated vasculitis. This is a report of ANCA-associated vasculitis with periaortitis following the second dose of COVID-19 mRNA vaccination, in which a 56-year-old man developed numbness and pain in his lower extremities three weeks after COVID-19 vaccination. Following the onset of sudden abdominal pain, a fluorodeoxyglucose-positron emission tomography scan revealed periaortic inflammation. Serum myeloperoxidase (MPO)-ANCA levels were significantly elevated, and renal biopsy revealed pauci-immune crescentic glomerulonephritis. Treatment with steroids and cyclophosphamide alleviated abdominal pain and numbness in the lower limbs, resulting in a decrease in MPO-ANCA titers. The side effects of COVID-19 vaccination are still unclear. This report has indicated that side effects associated with vaccines against COVID-19 may include ANCA-associated vasculitis. However, a causal relationship between COVID-19 vaccination and the development of ANCA-associated vasculitis has not yet been clearly demonstrated. COVID-19 vaccination will continue internationally, so it is necessary to accumulate similar case reports in the future.

## Introduction

Severe acute respiratory syndrome coronavirus type 2 (SARS-CoV-2) has caused a global pandemic resulting in many deaths. A vaccine was developed to contain the pandemic. This vaccine to prevent the development of coronavirus infection 2019 (COVID-19) was developed through large-scale clinical trials [1]. Common short-term adverse events that occur after vaccination include fever, fatigue, body pain, and headaches, which are known as transient responses [2]. Meanwhile, the long-term side effects are still unclear. However, as COVID-19 vaccines are being administered worldwide, rare side effects such as thrombosis and myocarditis have been reported. To our knowledge, immune-mediated diseases such as Guillain-Barré syndrome and idiopathic thrombocytopenic purpura are also among the long-term adverse effects of COVID-19 vaccines [3]. There has been an increase in reports of COVID-19 vaccinations being associated with the onset of autoimmune diseases, such as anti-neutrophil cytoplasmic antibody (ANCA)-associated vasculitis [4].

## Case presentation

A 56-year-old Japanese male with a history of gout presented to the emergency department complaining of upper abdominal pain and vomiting that persisted for 10 hours. He had no history of chronic inflammatory disease and was taking no medications. He had received the second dose of the BNT162b2 mRNA COVID-19 vaccine six weeks ago. While fever and myalgia persisted for a week following vaccination, all other symptoms had disappeared. Three weeks after the COVID-19 vaccination, numbness in the lower limbs persisted, accompanied by pain in the soles. He continued to have pain in his lower limbs and had difficulty walking until he came to our hospital.

**Figure 1:**
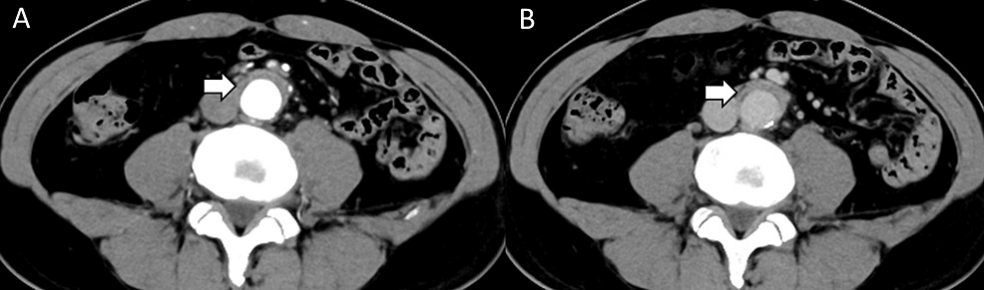
Periaortitis at the first visit. (A) Arterial and (B) equilibrium phase of contrast-enhanced computed tomography at the first visit: the abdominal aortic wall was thickened and periaortitis was suspected (arrow).

Blood test findings revealed no eosinophilia at the first visit, with creatinine levels of 1.07 mg/dL, blood urea nitrogen of 19.7 mg/dL, C-reactive protein (CRP) of 8.02 mg/dL, and a white blood cell count of 12,000/μL. His historical creatinine level was 0.83 mg/dL, and the current creatinine level was elevated above that known. Urine test findings showed urinary protein 2+ (0.53 g/g/Cr) and urinary occult blood 2+ (>40 red blood cells/high-power field). Abdominal computed tomography (CT) revealed localized arterial wall thickening of the abdominal aorta, and periaortic inflammation was suspected (Figure 1). The patient was instructed to take loxoprofen orally and revisit the hospital three days later.

**Figure 2:**
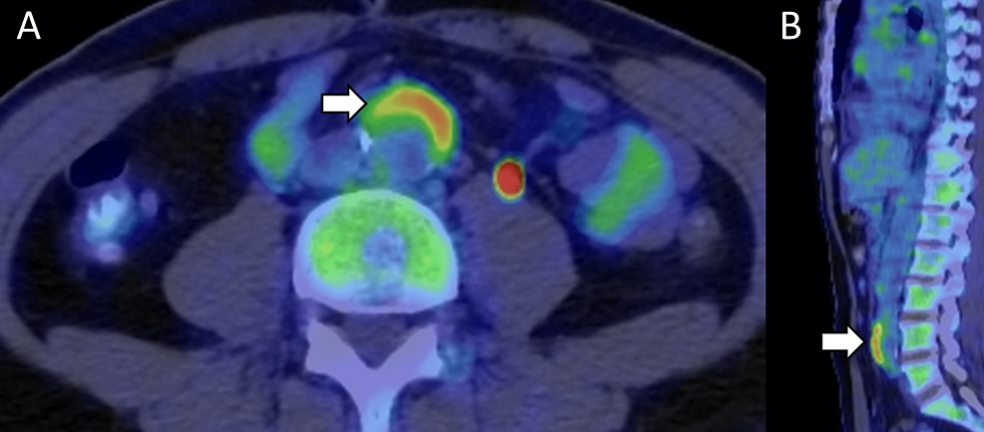
Periaortitis detected with fluorodeoxyglucose-positron emission tomography. (A) Axial and (B) coronal planes of fluorodeoxyglucose-positron emission tomography revealing periaortic inflammation (arrow).

On the second visit, although abdominal pain improved, pain and numbness in the lower limbs continued to increase, and the patient could no longer walk. Fluorodeoxyglucose-positron emission tomography (FDG-PET) scan was performed for the detailed examination of periaortic inflammation, which revealed wall thickening in the abdominal aorta below the renal artery bifurcation and abnormal FDG accumulation at the same site (Figure 2). FDG accumulation was confined to the periaortic area, and there was no FDG accumulation in the kidneys. ANCA-associated vasculitis was suspected as the cause of acute aortitis with microscopic hematuria and polyneuropathy, and, as a result, the patient was found to be positive with myeloperoxidase (MPO)-ANCA titers of 1,405.0 IU/mL. There was no remarkable abnormality in autoantibodies, complement levels, and other serological parameters, suggesting acute glomerulonephritis (Table 1).

**Table 1:** Laboratory tests before starting the treatment. AST: aspartate aminotransferase; ALT: alanine aminotransferase; MPO-ANCA: myeloperoxidase-anti-neutrophil cytoplasmic antibodies; PR3-ANCA: proteinase-3-anti-neutrophil cytoplasmic antibodies; RPR: rapid plasma reagin; TPHA: *Treponema pallidum* hemagglutination test

Laboratory test	Result	Normal values
White cell count	12,000	3,500–9,700/µL
Hemoglobin	10.3	13.6–18.3 g/dL
Platelets	38.8	14.0–37.9 × 10^4^/µL
Albumin	3.0	3.7–5.5 g/dL
AST	25	10–40 U/L
ALT	28	5–45 U/L
Total bilirubin	0.3	0.3–1.2 mg/dL
Urea nitrogen	19.7	8–20 mg/dL
Creatinine	1.07	0.65–1.09 mg/dL
C-reactive protein	8.02	<0.3 mg/dL
HbA1c	5.8	4.6–6.2%
IgG	2212	820–1,740 mg/dL
IgG4	87	11–121 mg/dL
MPO-ANCA	1,405.0	<3.4 IU/mL
PR3-ANCA	1.0	<1.9 IU/mL
RPR	Negative	Negative
TPHA	Negative	Negative
Blood culture	Negative	Negative
Uric blood	2+	Negative
Uric protein	2+	Negative
Uric protein/creatinine ratio	0.53	0–0.14 g/g/Cr

**Figure 3:**
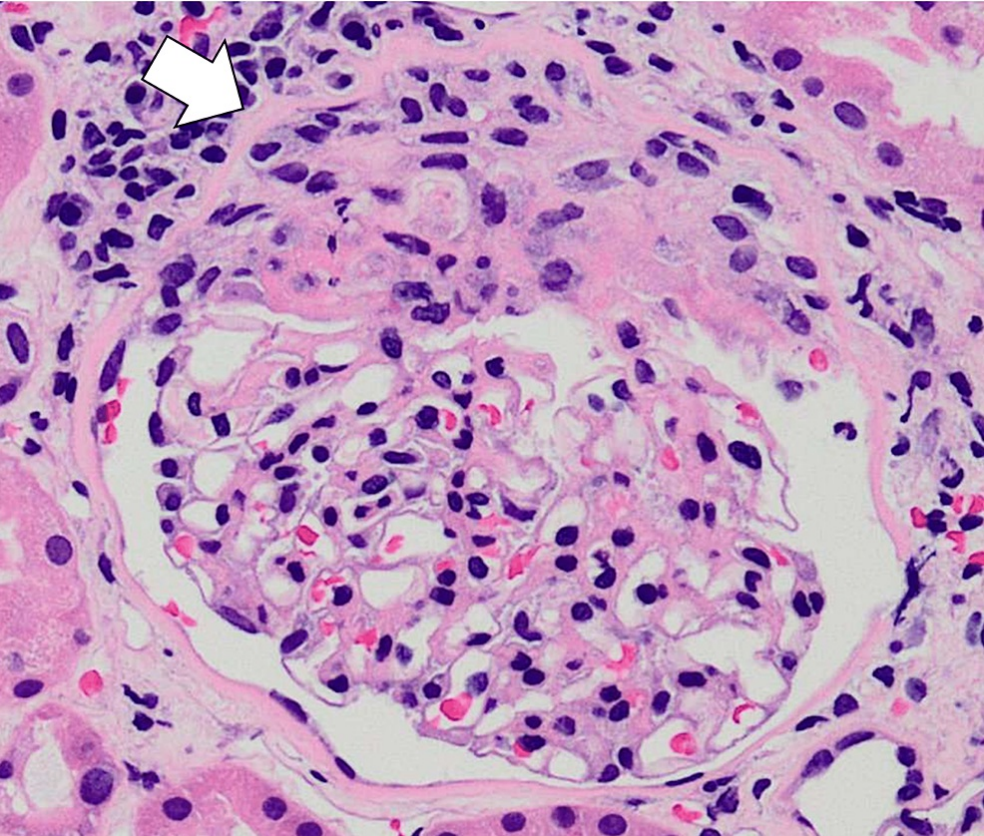
Histopathological findings obtained by renal biopsy. Renal biopsy confirmed cellular crescent formation with fibrinoid necrosis on hematoxylin and eosin staining (arrow, ×400).

Based on ANCA-associated vasculitis, the patient received steroid pulse therapy for three days via intravenous administration of methylprednisolone (1,000 mg/day). Then, at a dose of 1 mg/kg, prednisolone was administered orally (70 mg/day). The patient was temporarily transferred to another hospital to undergo a renal biopsy for the histological diagnosis of nephritis. Optical microscopy confirmed cellular crescent formation with fibrinoid necrosis in the collected glomerulus (Figure 3). Infiltration of mononuclear cells and polynuclear cells was observed in the renal interstitium, but infiltration of eosinophils and plasma cells was not conspicuous. An immunofluorescent antibody assay revealed no immune complex deposition in the glomerular basement membrane. As a result, the patient was diagnosed with ANCA-associated pauci-immune crescentic glomerulonephritis. Vasculitic neuropathy was suspected for numbness and pain in both lower limbs, and a manual neurological examination was performed. While the patellar tendon reflex was normal in both legs, the Achilles tendon reflex had disappeared in both legs. A vibration sense test on inner ankle joints revealed a slight disability with 12 seconds for the right foot and 11 seconds for the left foot, but there was no left-right difference. A nerve conduction study (NCS) showed a significantly weakened compound motor action potential of bilateral tibial nerves, indicating axonal injury. Therefore, the neurological symptoms in the lower limbs of the patient were diagnosed as peripheral neuropathy induced by ANCA-associated vasculitis.

**Figure 4:**
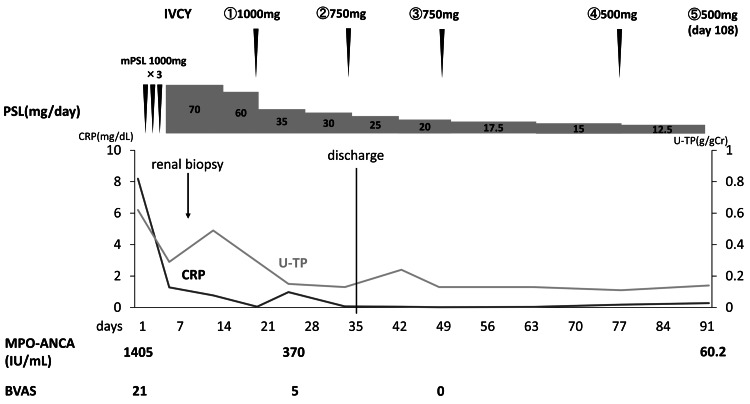
The time course of the administered dose of steroids and cyclophosphamide. IVCY: intravenous administration of cyclophosphamide; mPSL: methylprednisolone; PSL: prednisolone; CRP: C-reactive protein; U-TP: urinary total protein; MPO-ANCA: myeloperoxidase-anti-neutrophil cytoplasmic antibodies; BVAS: Birmingham Vasculitis Activity Score

**Figure 5:**
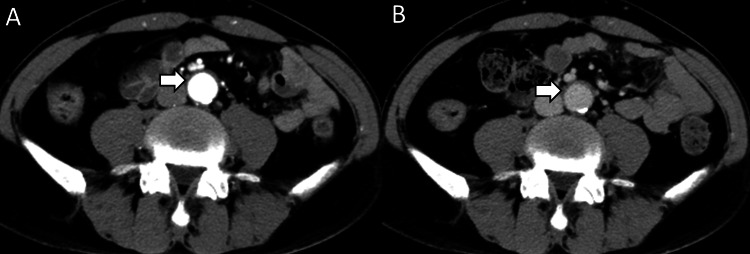
Contrast-enhanced computed tomography findings showing post-treatment periaortitis. (A) Arterial and (B) equilibrium phase of contrast-enhanced computed tomography five months after the start of treatment: the abdominal aortic wall thickening had disappeared (arrow).

Intravenous administration of cyclophosphamide was started at 15 mg/kg (1,000 mg each time) as the treatment for ANCA-associated vasculitis with crescentic glomerulonephritis and vasculitic neuropathy. Intermittent administration of cyclophosphamide every two weeks was planned, and the dose was reduced to 10 mg/kg (750 mg each time) after the second administration. The oral dose of prednisolone was reduced to 0.5 mg/kg (35 mg/day) after the start of cyclophosphamide administration, and a protocol was planned to reduce the oral dose of prednisolone by 5 mg every week thereafter. Thus, we chose rapidly progressive glomerulonephritis (RPGN)-based treatment for ANCA-associated vasculitis with periaortitis which is complicated by high levels of MPO-ANCA, nephropathy, and neuropathy. Blood CRP levels normalized after the second cyclophosphamide administration, and no red blood cell counts were detected in the urinary test. The urinary protein qualitative test was negative (0.13 g/g/Cr) after the third cyclophosphamide administration. As MPO-ANCA titers remained high (370.0 IU/mL), the oral administration of prednisolone was gradually reduced, and the intermittent administration of cyclophosphamide was stopped after a total of five times (Figure 4). Following the treatment, methotrexate was selected for maintenance immunosuppressive therapy because of the drug’s cost and administration. A combination of methotrexate and 8 mg prednisolone was orally administered, and MPO-ANCA continued to show a decreasing trend. Abdominal contrast-enhanced CT was repeated five months after the start of treatment. It was observed that among abnormal findings noted before admission, abdominal aortic wall thickening had disappeared (Figure 5). This finding supported the diagnosis of microscopic polyangiitis with large-vessel vasculitis even more strongly. To date, there have been no signs of recurrent vasculitis, and the patient has not been faced with the need to re-administer cyclophosphamide or rituximab.

## Discussion

In the Chapel Hill classification, ANCA-associated vasculitis is shown in the two types of systemic and organ-limited types, and it mainly causes small-vessel vasculitis [5]. The patient in our case was characterized by the coexistence of crescentic glomerulonephritis and mononeuritis multiplex, with positive MPO-ANCA. In particular, the absence of immune complex deposition and granulomatous lesions in the glomerular basement membrane in renal pathology strongly supported the diagnosis of microscopic polyangiitis. Furthermore, the patient developed abdominal pain, and FDG-PET revealed wall thickening with horseshoe-shaped FDG accumulation in the abdominal aorta, complicated by periaortic inflammation. Cases of ANCA-associated vasculitis with inflammation in the aorta and surrounding areas have been reported, which is known as ANCA-associated large-vessel disease [6]. Meanwhile, large-vessel lesions that occur in the presence of ANCA-associated vasculitis may overlap with diseases that cause large-vessel vasculitis, such as giant cell arteritis (GCA) and IgG4-related periaortitis. In addition to the aforementioned vasculitis, syphilis-associated aortitis and infective aortitis can cause periarteritis. However, opinions on the differentiation of ANCA-associated large-vessel diseases, including the interpretation of the disease spectrum, are divided [7]. In this case, serum IgG4 was normal; furthermore, blood culture and syphilis serum markers were negative. Because the patient had no history of headaches and did not undergo an arterial biopsy, it cannot be completely ruled out that GCA may have caused this periaortitis. However, because the patient had no history of inflammatory diseases, we assumed that each clinical symptom that developed during the acute course was related to ANCA-associated vasculitis.

The involvement of influenza vaccination in the relationship between vaccination and ANCA-associated vasculitis has long been known [8,9]. In patients genetically predisposed to autoimmune disease, theorized mechanisms such as molecular mimicry, polyclonal activation, or transient systemic inflammatory cytokines have been explained to be responsible for ANCA-associated vasculitis following influenza vaccination [10]. It has been suggested that a similar pathogenesis as in the case of the influenza vaccine may exist in the relationship between the COVID-19 vaccine and ANCA-associated vasculitis. Inflammatory cytokines activate neutrophil extracellular traps (NETs) formation and antibodies formation against MPO and proteinase-3. Furthermore, abnormal regulation of NETs may contribute to angiopathy and ANCA production [11].

Fortunately, even though ANCA-associated vasculitis is a treatable disease, it is necessary to continue to monitor whether the same treatment as before is effective even if the COVID-19 vaccine is involved in the onset. The cause-effect relationship with COVID-19 vaccines is unclear at present, but there are several reports of cases where patients newly developed ANCA-associated vasculitis or where ANCA-associated vasculitis recurred after COVID-19 vaccination [12-14]. In addition, the onset of ANCA-associated vasculitis has been reported for both mRNA and viral vector vaccines, but it is necessary to investigate further the type of vaccine that has a higher frequency of causing vasculitis. The onset of ANCA-associated vasculitis has often been observed after the second dose of the COVID-19 vaccine, but, in some cases, it may occur after the first vaccination [4]. The onset time varies, with onset occurring as soon as a few days after vaccination and as late as seven weeks [15]. A report on newly developed ANCA-associated vasculitis after COVID-19 vaccination from January to November 2021 accumulated 24 cases [4], with a median time from vaccination to onset of 14 days (2-28 days). In this case, the patient developed symptoms indicating neuropathy three weeks after receiving the second dose of the COVID-19 vaccine, similar to previous reports. This suggests that when investigating inflammatory diseases of unknown etiology, the inclusion of ANCA-associated vasculitis in the differential diagnosis may be diagnostically helpful, depending on the timing of COVID-19 vaccination.

A limitation of this case report is that we cannot prove a causal relationship between the COVID-19 vaccine and ANCA-associated vasculitis. However, the temporal relationship suggests that the COVID-19 vaccine may have been causative. Accumulation of similar cases is needed in the future to clarify the cause of this disease.

## Conclusions

We reported a case of an adult male who developed ANCA-associated vasculitis after COVID-19 vaccination. However, there is no clear evidence that ANCA-associated vasculitis is a side effect of COVID-19 vaccination. Nevertheless, there have been several reports of ANCA-associated vasculitis that developed after COVID-19 vaccination. Knowledge of autoimmune diseases that develop after COVID-19 vaccination is still limited, so it is important to accumulate such case reports in the future. In addition, when we encounter unexplained inflammatory diseases in real-world clinical practice, considering the association with the COVID-19 vaccine may contribute to a correct diagnosis. In such cases, it is worth checking the timing of COVID-19 vaccination, the type of vaccine, and the number of vaccine doses.

## References

[1] Polack FP, Thomas SJ, Kitchin N (2020). Safety and efficacy of the BNT162b2 mRNA Covid-19 vaccine. N Engl J Med.

[2] Shimabukuro TT, Cole M, Su JR (2021). Reports of anaphylaxis after receipt of mRNA COVID-19 vaccines in the US-December 14, 2020-January 18, 2021. JAMA.

[3] Ibrahim H, Alkhatib A, Meysami A (2022). Eosinophilic granulomatosis with polyangiitis diagnosed in an elderly female after the second dose of mRNA vaccine against COVID-19. Cureus.

[4] Prabhahar A, Naidu GS, Chauhan P (2022). ANCA-associated vasculitis following ChAdOx1 nCoV19 vaccination: case-based review. Rheumatol Int.

[5] Jennette JC, Falk RJ, Bacon PA (2013). 2012 revised International Chapel Hill Consensus Conference Nomenclature of Vasculitides. Arthritis Rheum.

[6] Chirinos JA, Tamariz LJ, Lopes G, Del Carpio F, Zhang X, Milikowski C, Lichtstein DM (2004). Large vessel involvement in ANCA-associated vasculitides: report of a case and review of the literature. Clin Rheumatol.

[7] Coattrenec Y, Muller YD, Spoerl D, Lobrinus JA, Seebach JD (2021). Prevalence of large vessel vasculitis in ANCA-associated vasculitis: a retrospective cohort study. Rheumatol Int.

[8] Watanabe T (2017). Vasculitis following influenza vaccination: a review of the literature. Curr Rheumatol Rev.

[9] Duggal T, Segal P, Shah M, Carter-Monroe N, Manoharan P, Geetha D (2013). Antineutrophil cytoplasmic antibody vasculitis associated with influenza vaccination. Am J Nephrol.

[10] Jeffs LS, Nitschke J, Tervaert JW, Peh CA, Hurtado PR (2016). Viral RNA in the influenza vaccine may have contributed to the development of ANCA-associated vasculitis in a patient following immunisation. Clin Rheumatol.

[11] Nakazawa D, Masuda S, Tomaru U, Ishizu A (2019). Pathogenesis and therapeutic interventions for ANCA-associated vasculitis. Nat Rev Rheumatol.

[12] Shakoor MT, Birkenbach MP, Lynch M (2021). ANCA-associated vasculitis following Pfizer-BioNTech COVID-19 vaccine. Am J Kidney Dis.

[13] Dube GK, Benvenuto LJ, Batal I (2021). Antineutrophil cytoplasmic autoantibody-associated glomerulonephritis following the Pfizer-BioNTech COVID-19 vaccine. Kidney Int Rep.

[14] Gillion V, Jadoul M, Demoulin N, Aydin S, Devresse A (2021). Granulomatous vasculitis after the AstraZeneca anti-SARS-CoV-2 vaccine. Kidney Int.

[15] Chen CC, Chen HY, Lu CC, Lin SH (2021). Case report: anti-neutrophil cytoplasmic antibody-associated vasculitis with acute renal failure and pulmonary hemorrhage may occur after COVID-19 vaccination. Front Med (Lausanne).

